# Dentition Status and Oral Health Practice among Hearing and Speech-Impaired Children:A Cross-sectional Study

**DOI:** 10.5005/jp-journals-10005-1091

**Published:** 2010-04-15

**Authors:** Suma G, Usha Mohan Das, Akshatha BS

**Affiliations:** 1 Senior Lecturer, Department of Pedodontics and Preventive Dentistry, VS Dental College and Hospital, Bengaluru, Karnataka, India; 2 Professor and Head, Department of Pedodontics and Preventive Dentistry, VS Dental College and Hospital, Bengaluru, Karnataka, India; 3 Postgraduate Student, Department of Pedodontics and Preventive Dentistry, VS Dental College and Hospital, Bengaluru, Karnataka, India

**Keywords:** Hearing-impaired, Speech-impaired, Dental caries, Disabled.

## Abstract

The main aim of this study was to assess the oral health status and oral hygiene practices in children with impaired hearing and speech. A total of 76 children in the age group of 5 to 18 years of both sexes were surveyed and information about their oral hygiene practices, previous dental visit and oral health knowledge were obtained through a questionnaire. Around 61% of the children had never visited a dentist, 82.89% and 17.11% of them brushed once and twice daily respectively. More than 90% of them cared about their teeth as much as any other part of the body. 42% of the children had dental caries, and gingivitis was seen in 35% of the children and malocclusion in 19% of them.

## INTRODUCTION

Children and adolescents with disabilities appear to have poorer oral health than their nondisabled counterparts. Oral health is an important aspect of health for all children, and is all the more important for children with special health needs. Because oral hygiene affects one’s esthetics and communication, it has strong biological, psychological and social projections.^1^ Variable access to dental care, inadequate oral hygiene and disability related factors may account for the differences.^2^ The type of dental care received may be determined more by the disability than the oral condition, compounding the chronicity of dental disease. Although there have been a number of studies concerning the oral health of children, in general, there have been relatively few investigations of the oral conditions of the disabled children.^3^

It is believed that the number of handicapped individuals is increasing in proportion to the general population.^4^ Dental care is the most common unmet health care need of disabled children.^5^

Studies have shown that all the common dental disorders affecting the normal population are to be seen in handicapped people (Franks and Winter, 1974). In the latter, these disorders may occur either more often with increased severity or at a younger age than might be considered usual for normal individuals. Accumulation of bacterial plaque has been identified as the main cause, of the two most common dental diseases (caries and periodontal diseases) are seen in handicapped and normal children (Bear and Benjamin, 1974).^6,7^

Data concerning the oral health condition of handicapped people are scarce. Most reports are based on an examination of small number of individuals, subjects with widely differing ages or with different handicapping conditions. Reports of oral condition restricted to deaf and dumb children in specific are lacking.

Children with hearing impairement constitute one of the major population groups of handicapped children. According to National Sample Survey Organisation (NSSO) of India in 2002, 0.4% of 1065.40 million children suffered from hearing impairement. Earlier studies on their oral health status reported poor oral hygiene and low utilization of dental services.^8,9^

Dental care is not a priority to families of the multi-disabled child. Improvement in oral health status can be achieved through on-site oral health care. More awareness of the dental care needs of these children is necessary.

Health care providers must have unique communication skills to deal with the special needs of deaf children. Programs designed to improve knowledge, attitude and behavior should be innovative to meet the special needs of this population.^9^

The aim of this study was to assess the oral health status and oral hygiene practices in children with impaired hearing and speech.

## METHODS

The study was conducted in RV Integrated School for the Disabled which constitutes 76 speech and hearing-impaired children. After selection of the school, parents of the children were informed about the examination. The children were examined at the institution by using a mouth mirror, probe and daylight in accordance with the WHO survey recommendations, and caries, periodontal disease, malocclusion, oral health status and treatment needs were recorded on the simplified WHO oral health criteria and assessment form. Each examination took about 10 to 15 minutes.

Sociodemographic information, previous dental visits, toothbrushing and snacking habits, perceived dental problems and willingness to have dental check-up were obtained from questionnaires completed under the supervision of the parent/caregiver.

## RESULTS

**Fig. 1:**
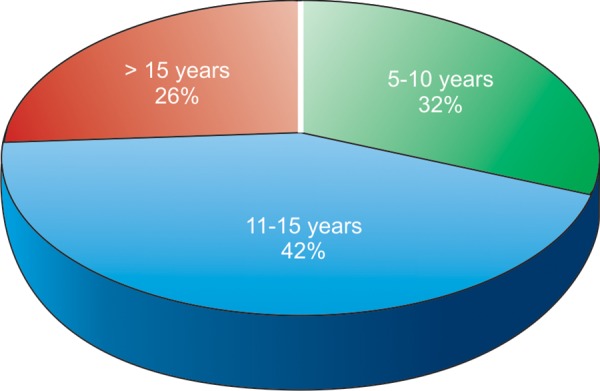
Sample distribution according to age groups

**Fig. 2:**
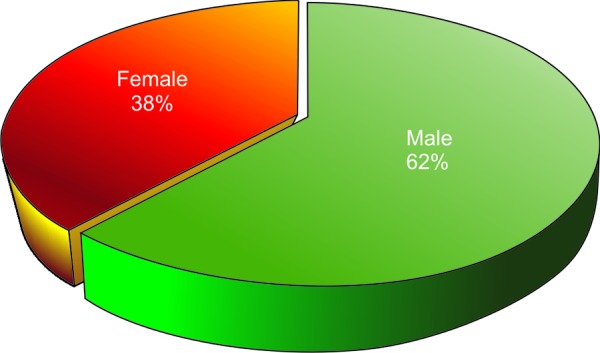
Sample distribution according to gender

A total of 76 children, 47 males and 29 females, aged 5 to 18 years took part in the study (Tables 1 and 2, Figs 1 and 2). Majority of them had never visited the dentist (80.26%). Among those who visited the dentist most of them visited when they had dental pain (14.47%) and extraction followed by restorations was the most common treatment done. Around 71.05% and 56.58% of the children felt that fizzy drinks and sweets did not affect the teeth adversely. About 71% of them reported that brushing teeth prevent dental decay and more than 90% of them cared about their teeth as much as any other part of their body. 82.89% of them brushed once daily and 17.11% of them brushed twice daily, 42.11% of them brushed for about 2 minutes, 55.26% of the children were advised by parents to brush properly (Table 3).

**Table 1:** **Table 1:** Distribution of the study sample according to age group

*Age group (years)*		*n*		*%*	
5-10		24		31.58	
11-15		32		42.11	
>15		20		26.32	
*Total*		76		100	

**Table 2:** **Table 2:** Gender distribution in the study sample

*Gender*		*n*		*%*	
Male		47		61.84	
Female		29		38.16	
*Total*		76		100	

**Table 3:** **Table 3:** Oral health knowledge and practices

		*n*		*%*	
*Brushing teeth*					
Once per day		63		82.89	
Twice per day		13		17.11	
*Cleaning teeth*					
Brush + Toothpaste		74		97.37	
Finger + Toothpowder		2		2.63	
*Time of brushing*					
Morning		63		82.89	
Morning and night		13		17.11	
*Duration of brushing*					
2 minutes		32		42.11	
> 2 minutes		21		27.63	
*Parents*					
Do not watch but advise me		42		55.26	
Never cared		14		18.42	
*Visiting the dentist*					
I never visited the dentist		61		80.26	
When I have dental pain		11		14.47	
*Last visit to the dentist*					
Haven’t visited		61		80.26	
Last 1-2 years		8		10.53	
*Treatment*					
No treatment		65		85.53	
Extraction		6		7.89	
Restoration		5		6.58	

**Table:** 

*Question*		*Yes*				*No*		
		*n*		*%*		*n*		*%*
Sweets affect the teeth adversely		33		43.42		43		56.58
Fizzy drinks affect the teeth adversely		22		28.95		54		71.05
Brushing teeth prevent dental decay		54		71.05		22		28.95
You care about your teeth as much as		71		93.42		5		6.58
other parts of your body								

Among, the 76 children enrolled in the study, the caries prevalence was 42% with the D/d component higher than 72% (Tables 4 and 5). 87% of the children required single surface or double surface restorations, the remaining were indicated for pulp therapy. Gingivitis was seen in 35% of the children with bleeding gums and calculus who required oral prophylaxis. The study showed that 19% of the subjects had malocclusion which constituted anterior openbite seen in 3%, crowding in 11% and class II malocclusion seen in 3%. Fractured anterior teeth were seen among 3.9% of the children examined.

We observe that there is no significant difference between age groups with respect to the proportion of dental caries in the study population (p > 0.05).

## DISCUSSION

Majority of the children had never visited the dentist for a check-up or treatment. These findings are similar to findings in previous studies on the disabled. This could be due to low priority of parents on oral health care.

There are many other difficulties faced by deaf children, leading to inequalities when they are compared with hearing people to access oral health care more so in young children with speech and hearing impairement.^10^

Very few children knew the harmful effects of sweets and fizzy drinks on their teeth but were aware that brushing their teeth daily prevented dental decay. These results are similar to the study conducted by Oredugba FA (2004) where only 8% of them gave correct answers to causes of tooth decay.^9^

A review article by Nunn (1987) states the dental health of children with a handicap is similar to that of ‘normal’ children. This article mentions that children with a handicapping condition have more untreated decay and have had more teeth extracted compared to their ‘normal’ counterparts.^11^

Caries prevalence in the present study was 42% and demonstrated a higher prevalence in the age group of 11 to 15 years in accordance with the study conducted by Rao et al (2001) in Mangalore, showed caries prevalence of 46% in 5 to 9 years and 48% in 10 to 14 years age group of children. In the present study, the results showed that the girls’ oral hygiene was better than those of boys similar to the findings by Rao et al.^12^

In another study conducted by Gupta et al (1993) in Calcutta, the caries prevalence was 55.9%.^13^ Damle et al (1995) reported caries prevalence of 78.3%. In general, it was observed that all the children had a very high decayed (D) component as compared to the missing (M) and filled (F) components.^14^ In our study also a very high decayed component was seen similar to the study conducted by Damle et al.

In a study conducted by Kumar et al (2003) in Bel-gaum, Karnataka, it was observed that periodontal health was generally poor in all the children. The results of our study showed 35% of the children had bleeding gums or calculus.^15^

**Table 4:** **Table 4:** Gender-wise distribution of dental caries

*Gender*		*Caries free*				*Caries present*				*Total*	
		*n*		*%*		*n*		*%*			
Male		29		66		18		56		47	
Female		15		34		14		44		29	
Total		44		100		32		100		76	

**Table 5:** **Table 5:** Age-wise distribution of dental caries

*Age group* *(years)*		*n*		*Mean*		*Std dev*		*Min*		*Max*		*Kruskal-* *Wallis* *Chi-sq*		*p-* *value*	
5-10		24		0.42		0.50		0		1		0.781		0.677	
11-15		32		0.38		0.49		0		1					
>15		20		0.50		0.51		0		1					

## CONCLUSION

There was a high prevalence of dental caries and the need for restorative care among the children of this study. There is a need for implementation and evaluation of a long-range public dental health care plan for children with disabilities. In view of the findings of this study, frequent maintenance visits and oral hygiene interventions, including prophylaxis, restorative care and evaluation of the oral tissues, is recommended. It leads to the conclusion that preventive care has to be implemented in this population to further prevent dental caries and periodontal diseases. There is a need for comprehensive oral health care programs for these children.
